# Regulation of Tumor and Metastasis Initiation by Chemokine Receptors

**DOI:** 10.7150/jca.72331

**Published:** 2022-08-27

**Authors:** Anthony DiNatale, Maria Sofia Castelli, Bradley Nash, Olimpia Meucci, Alessandro Fatatis

**Affiliations:** 1 Department of Pharmacology and Physiology, Drexel University College of Medicine, Philadelphia, PA 19102, USA.; 2 Present Address: Janssen Oncology, Spring House, PA, USA.; 3 Present address: Perelman School of Medicine, University of Pennsylvania, Philadelphia, PA 19104, USA.; 4 Program in Immune Cell Regulation & Targeting, Sidney Kimmel Cancer Center of Thomas Jefferson University, Philadelphia, PA 19107, USA; 5 Program in Translational and Cellular Oncology, Sidney Kimmel Cancer Center of Thomas Jefferson University, Philadelphia, PA 19107, USA.

**Keywords:** Chemokines, Initiation, Metastasis, Pluripotency

## Abstract

Tumor-initiating cells (TICs) are a rare sub-population of cells within the bulk of a tumor that are major contributors to tumor initiation, metastasis, and chemoresistance. TICs have a stem-cell-like phenotype that is dictated by the expression of master regulator transcription factors, including OCT4, NANOG, and SOX2. These transcription factors are expressed via activation of multiple signaling pathways that drive cancer initiation and progression. Importantly, these same signaling pathways can be activated by select chemokine receptors. Chemokine receptors are increasingly being revealed as major drivers of the TIC phenotype, as their signaling can lead to activation of stemness-controlling transcription factors. Additionally, the cell surface expression of chemokine receptors provides a unique therapeutic target to disrupt signaling pathways that control the expression of master regulator transcription factors and the TIC phenotype. This review summarizes the master regulator transcription factors known to dictate the TIC phenotype, along with the complex signaling pathways that can mediate their expression and the chemokine receptors that are most upstream of this phenotype.

## Introduction

Tumor-initiating cells (TICs) are a small population within tumors that possess stem-like properties, permitting them to initiate tumor growth as single cellular units. Their presence has been confirmed in many cancer types, including prostate, breast, colon, ovarian, and melanoma, among others 1-5. Solid tumors can be treated with local modalities like surgery and radiation paired with systemic therapies, but the risk of distant dissemination - or metastasis - remains a major concern. Patients with metastatic lesions may achieve a temporary decrease in tumor burden with systemic treatment, but drug resistance will eventually emerge and ultimately lead to patient death 6. As a result, most cancer deaths are not directly caused by the primary tumor, but rather are due to metastatic disease 7. Metastasis-initiating cells (MICs) develop secondary lesions in target organs. MICs can withstand the shear forces existing in the blood vessels of the systemic circulation and disseminate to secondary sites, where they successfully colonize new tissue microenvironments and grow into metastases. Therefore, in this review we use MICs to broadly indicate cells that retain the TIC phenotype and exhibit additional properties that increase their malignancy and the potential for a lethal clinical outcome.

Tumor recurrence is a consequence of incomplete eradication of cancer cells and leads to the regrowth of existing lesions and the development of new ones. MICs have also been associated with drug resistance and are therefore well positioned to survive and repopulate the tumors after the ablation of most of the drug-sensitive cells by systemic therapies. In addition, clinical studies reveal that aggressive malignancies can be identified by gene expression profiles associated with stem cell pathways, and these same genes are associated with poor response to therapy for a wide range of tumors 8.

MICs have features that distinguish them from differentiated, non-stem-like cancer cells. They divide in an asymmetric fashion, giving rise to one MIC and one differentiated cell. A differentiated cell is only further capable of producing differentiated progeny harboring a higher proliferative rate and thus are responsible for constituting the majority of tumor cells. The daughter MIC then undergoes the same process of asymmetric division, generating one differentiated daughter cell and another MIC, resulting in self-renewal and maintenance of a steady-state population of MICs within the bulk tumor population 9. Label-retaining studies provides evidence for asymmetric division as a way to self-renewal. When cellular DNA is labeled, the label is normally diluted as cells symmetrically divide, until the label can no longer be detected. In asymmetric division, the labeled DNA of MICs is retained by the MIC progeny and in gastrointestinal cancers, bromodeoxyuridine DNA labeling was elegantly used to identify label-retaining cells exhibiting a TIC phenotype 10. MICs also have slow proliferation rates, which partially explains their resistance to anti-proliferative therapies including chemotherapies and radiotherapies 11. These cells concurrently exploit other mechanisms of treatment resistance, including high expression of multidrug resistance proteins and enhanced cell signaling to protect against DNA damage-induced cell death 12, which underscores their involvement in tumor relapse. While treatment with first-line chemotherapy will eliminate most susceptible non-stem-like cancer cells, MICs will ultimately survive. These cells can then undergo genetic alterations, expand, and differentiate, leading to the development of metastatic lesions that result in patient death 13.

MICs are difficult to fully identify and characterize due to their low abundance. The surface receptor CD44 has been established as a stemness marker across various cancer types 14. Another stemness marker for multiple cancer types is CD133, for which an important role has been proposed in pancreatic cancer 15 and in prostate cancer 16. A greater understanding of the surface receptors expressed by MICs will provide not only opportunities for their isolation and characterization but will also help identifying novel therapeutic targets.

Cancer cell stemness is regulated by several pluripotency-associated transcription factors, but it is not fully known how the expression of these genes is regulated in MICs. Increasing evidence suggests this is mediated by a complex network of cell signaling pathways that differs by cancer type. Recent studies suggest that these pathways may be regulated by signaling via various chemokine/chemokine receptor pairs on MICs, which may provide a novel strategy to target the establishment and maintenance of the TIC phenotype.

Here, we describe some of the signaling pathways reportedly involved in the transcriptional regulation of pluripotency master regulators in MICs. We then review the chemokine receptors found to regulate the expression of pluripotency-associated transcription factors and discuss the tumor models in which their role in this process has been reported.

## Pluripotency master regulator transcription factors

The TIC phenotype is regulated by several pluripotency-associated transcription factors, including octamer binding transcription factor 4 (OCT4), sex determining region Y - related high mobility group box 2 (SOX2) and nanog homeobox (NANOG). Upregulation of these genes helps cancer cells acquire stemness properties, and thus, they are considered critical regulators of self-renewal and pluripotency that mediate tumor proliferation and differentiation 17-19.

### OCT4a

OCT4 (also known as OCT3) is a transcription factor encoded by the *Pou5f1* gene. In humans, this gene can generate three isoforms by alternative splicing, known as OCT4a, OCT4b and OCT4b1 20. OCT4a, normally referred to as OCT4, has been established as a marker for human pluripotent embryonic stem cells. OCT4 is a transcription factor that maintains the pluripotent state during embryonic development, and its loss leads to stem cell differentiation 20,21. The other isoforms, OCT4b and OCT4b1, may play a role in the biologic response of cells to stress 22,23. Accumulating evidence suggests that OCT4 helps maintain stemness features in cancer, thus playing a major role in self-renewal, cell survival, metastasis and drug resistance in MICs through the regulation of its target genes 17. OCT4 is overexpressed in MICs in various cancers and its high expression correlates with poor clinical outcomes 24. OCT4 also forms heterodimers with other transcription factors like SOX2, which can occur via their unique interactions with DNA. The OCT4 POU domain interacts with the major groove of the DNA, whereas a SOX2 high-mobility group (HMG) domain interacts with the minor groove of DNA 25, allowing OCT4 and SOX2 to dimerize with each other. This interaction drives the transcription of their target genes, which also includes SOX2, OCT4, and NANOG 26.

### SOX2

SOX2 is a transcription factor in the Sry-related HMG box (SOX) family of proteins, which bind to specific DNA sequences via a highly conserved HMG domain. SOX2 regulates the pluripotency and self-renewal of stem cells during embryogenic development and adult tissue regeneration 27. It is also involved in tumorigenesis in a wide range of cancers, including those of the breast, prostate, brain, lung, kidney, and skin. SOX2 regulates its target genes to promote cancer cell growth, invasion, migration, metastasis, and chemoresistance 28. Overexpression of SOX2 correlates with a stem-like phenotype in cancer and has been implicated in poor survival rates of cancer patients 28.

### NANOG

NANOG is a transcription factor with a DNA-binding homeodomain that helps maintain pluripotency in embryonic stem cells and is downregulated upon differentiation 29. As mentioned, NANOG expression is regulated by the OCT4/SOX2 complex, but NANOG can also be maintained without OCT4 involvement through activation by FoxD3 30. Like OCT4 and SOX2, NANOG can promote cell survival, anti-apoptotic signaling, migration, invasion, and chemoresistance, and is overexpressed in various cancers, including breast, ovarian, melanoma, and others 31.

Although there is a wide understanding of the role of the master regulators OCT4, SOX2, and NANOG in embryonic stem cells, it is crucial that we establish the same knowledge in MICs. While it is now evident that high expression of these pluripotency-associated transcription factors indicates a poor prognosis across cancer types, the mechanistic underpinning of this observation remains limited. By elucidating the signaling pathways that are hijacked by MICs to activate OCT4, SOX2, and NANOG, we will expand the number of targetable options for treating or controlling metastatic disease.

## Signaling pathways that activate the pluripotency master regulator transcription factors

**Figure 1:**
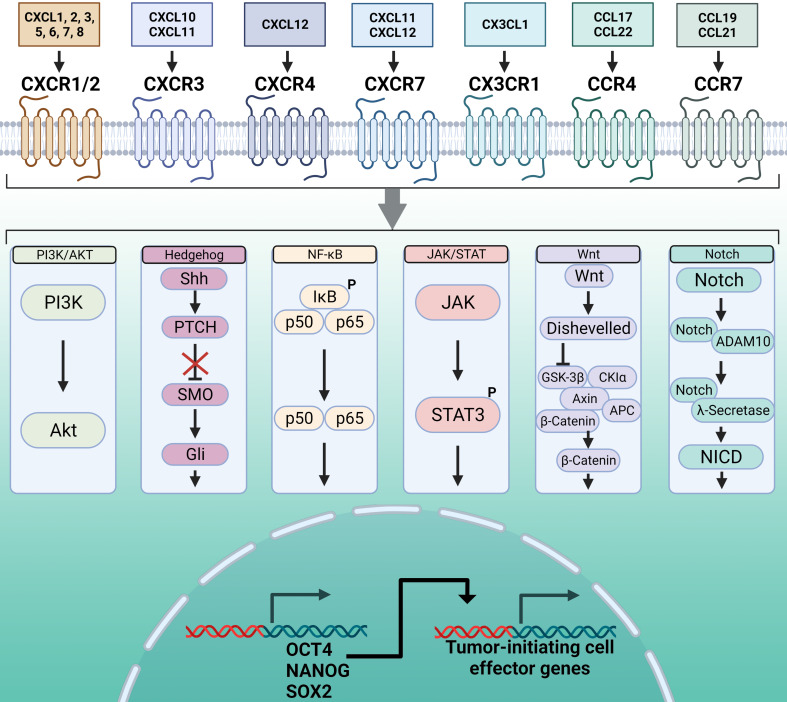
** Overview of the pathways mediating chemokine receptor-driven activation of master regulator transcription factors and the TIC phenotype.** At the cell surface, chemokines interact with chemokine receptors, which are G protein-coupled receptors, to activate their downstream cell signaling programs. Chemokine receptors can activate multiple signaling pathways, based on cell type and phenotype. Of these signaling pathways, the most widely characterized are PI3K/AKT, Hedgehog, NF-κB, JAK/STAT, Wnt, and Notch. A consequence of activation of these signaling pathways through chemokine binding is activation of the master regulator transcription factors OCT4, NANOG, and SOX2. These master regulator transcription factors support the TIC phenotype through chromatin interactions and activation of their downstream effector genes. Created with Biorender.com.

Like the master regulators OCT4, SOX2, and NANOG, multiple molecular signaling pathways that regulate cellular pluripotency are also dysregulated in cancer (**Figure 1**).

### PI3K/AKT pathway

The phosphatidylinositol-3-kinase (PI3K)/AKT pathway regulates many physiologic cellular processes, such as cell growth, proliferation, and survival. As these processes are also crucial for tumorigenesis, the PI3K/AKT pathway is often affected by genomic aberrations in cancer, thus contributing to tumor initiation and progression 32.

The PI3K/AKT pathway can regulate the expression of several pluripotency-associated transcription factors. Several studies have suggested that the PI3K/AKT pathway cooperatively interacts with SOX2 in cancer. Specifically, AKT kinase activity drives SOX2 nuclear localization and stabilization, and SOX2 supports PIK3CA gene expression that leads to further AKT activation 33. In breast cancer, SOX2 appears to act as a functional downstream AKT target, with a direct physical interaction between the two proteins. AKT phosphorylation of SOX2 can modulate SOX2 nuclear entry and its action on target genes, while inhibition of AKT leads to SOX2 retention in the cytosol and promotes its degradation via the proteasome 34. Similar studies in nasopharyngeal carcinoma and esophageal cancer show that PI3K/AKT signaling regulates SOX2 expression. AKT in nasopharyngeal carcinoma regulates the expression of the cell cycle regulator p27, which in turn modulates SOX2 expression 35, and AKT in esophageal cancer promotes overexpression of SOX2 and protects SOX2 from proteasomal degradation 36. In prostate cancer, PI3K/AKT is a key signaling pathway that maintains the SOX2/OCT4-overexpressing TIC population *in vitro* and *in vivo* 37. Indeed, prostate cancer MICs can upregulate SOX2 protein via TGF‐α-mediated activation of the EGFR/PI3K/AKT pathway 38.

Multiple cancer types also show a reciprocal relationship between the PI3K/AKT pathway and OCT4. In embryonal carcinoma, AKT phosphorylates OCT4, which stabilizes OCT4 and facilitates its nuclear localization and interaction with SOX2 39. This in turn promotes the transcription of the core stemness genes OCT4 and NANOG. Consequently, the levels of phosphorylated OCT4 in these cells positively correlated with tumorigenic potential in a xenograft model and resistance to apoptosis. Phosphorylation of OCT4 by AKT also led to dissociation of OCT4 from the AKT1 promoter, which activated AKT1 transcription and promoted cell survival 39. Similarly, AKT phosphorylation of OCT4 was associated with the proliferation of glioblastoma cancer cells *in vitro*, and when the authors attenuated AKT activation using the aryl hydrocarbon receptor ligand ITE, they found reduced proliferation of glioblastoma spheroids 40. OCT4 expression may also be regulated by nitric oxide (NO), a molecule that is upregulated in lung cancer and affects several cellular processes. In the absence of NO, OCT4 forms a molecular complex with the protein caveolin-1 that promotes its proteasomal degradation, but in the presence of NO, caveolin-1 is phosphorylated by AKT, which disrupts its complex with OCT4 and prevents OCT4 degradation *in vitro* 41. In breast cancer, PD-L1 helps maintain a stemness phenotype in MICs *in vitro* and in a mouse xenograft model via PI3K/AKT signaling that leads to phosphorylation of OCT4 and subsequent regulation of OCT4 and NANOG expression 42. Notably, PD-L1 knock-down inhibited the phosphorylation of AKT and OCT4, which was associated with decreased stemness and self-renewal of breast cancer with TIC phenotype both in tumorspheres *in vitro* and in an *in vivo* extreme limiting dilution assay. Finally, in lung cancer cells an activated AKT-ubiquitin proteasome degradation pathway downregulated OCT4 and NANOG as well as other TIC markers *in vitro*, further suggesting the PI3K/AKT pathway regulates these transcription factors 43.

### Hedgehog pathway

The Hedgehog (Hh) pathway is essential for normal embryonic development but also regulates genes involved in various processes in cancer, including differentiation, proliferation, and carcinogenesis 44.

The Hh pathway plays a role in the maintenance and function of stemness features in MICs, including tumor initiation and resistance to chemotherapy and radiotherapy 44. The stemness phenotype induced by Hh signaling seems to be driven by transcriptional regulation of pluripotency-associated genes. In melanoma, Hh signaling increased SOX2 expression and thereby supported self-renewal and tumorigenicity, whereas SOX2 silencing or depletion blocked cell growth, sphere formation, and the ability to initiate tumors in xenograft models 45. In pancreatic cancer, cooperation of Hh and EGFR signaling induced high-level expression of SOX2 that promoted both tumor initiation and tumor growth 46. As before, these effects were mitigated by knock-down of Hh/EGFR cooperative genes, including Sox2, Jun, and Cxcr4. Additionally, pancreatic cells with a TIC phenotype have been reported to show high levels of Hh activity that is thought to drive stemness features. Indeed, inhibition of Hh signaling in these cells *in vitro* decreased their expression of OCT4 and NANOG, reduced spheroid proliferation, and induced cellular apoptosis 47. In glioma-initiating cells (GICs), Hh signaling regulated SOX2 expression by helping Gli2 bind to the SOX2 promoter, and this pathway as well as GIC properties was mitigated by knock-down of urokinase receptor (uPAR) and cathepsin B 48. In another study of gliomas, treatment with an inhibitor of the Hh pathway decreased expression of OCT4, SOX2, and NANOG in GICs 49, further supporting that this pathway regulates stemness. Similarly in breast cancer, Hh signaling can be enhanced via expression of tetraspanin 8 and LncRNA-Hh, which increases the expression of OCT4, SOX2, and NANOG and leads to enhanced self-renewal and oncogenicity 50,51. Collectively, it is evident that Hh signaling regulates pluripotency genes in numerous cancer types.

### NF-kB pathway

The nuclear factor-κB (NF-κB) family of transcription factors plays crucial roles in cell survival, proliferation, apoptosis regulation, immunity, and inflammation 52. The NF-κB pathway controls the transcription of a large subset of genes, including central components of the immune response such as cytokines, as well as regulators of apoptosis, proliferation, and development 53. Thus, dysregulation of this pathway has been associated with several diseases, including immune disorders and cancer 52.

Activation of the NF-κB pathway has been associated with tumor initiation and progression through supporting processes such as cell survival, invasion, metastasis, angiogenesis, and apoptosis resistance. NF-κB activation is enhanced in MICs from various tumor types, including glioblastoma, prostate, breast, and ovarian 54. In lung cancer, inhibiting NF-κB signaling using an IκB kinase inhibitor mitigated stemness features 55. This was mediated by the reduced transcription of NF-κB target genes, which include the pluripotency genes OCT4, SOX2, and NANOG as well as genes involved in EMT and resistance to apoptosis. Similarly in breast cancer, the NF-κB pathway regulates the expression of OCT4, SOX2, and NANOG via upregulation of components of the NF-κB signaling pathway, including NIK 56,57. As expected, breast tumor growth was delayed by inhibiting NF-κB in a transgenic mouse model as well as knocking down NIK in breast cancer cells used for mouse xenograft studies. In castration-resistant prostate cancer cells, this pathway helps regulate the expression of OCT4, SOX2, and NANOG by activating a p65-mediated feed forward circuit that leads to the phosphorylation of IκBα 58. This pathway was also observed in a mouse xenograft model as well as human prostate tumors, further suggesting the importance of NF-κB signaling to stemness phenotypes. In ovarian cancer cells, cisplatin treatment enriched for a population with stemness features and induced NF-κB translocation to the nucleus, most prominently in cells with high OCT4 expression. Enhanced nuclear co-localization of NF-κB with OCT4 led to the increased expression of OCT4, SOX2, and NANOG and induced a TIC phenotype in these cells 59. The NF-κB pathway also regulates OCT4 expression in colorectal cancer via the signaling of pro-inflammatory cytokines interleukin-6 (IL-6) and TNFα, which induces stem-like behavior 60.

### JAK/STAT pathway

The Janus kinase/signal transducers and activators of transcription (JAK/STAT) pathway is involved in many important biological processes, including cell proliferation, differentiation, apoptosis, and immune regulation. The main components of this pathway are a tyrosine kinase-related receptor, the tyrosine kinase JAK, and the transcription factor STAT.

Aberrant JAK/STAT activation has been detected in many tumors, including by JAK2 mutations that lead to STAT3 activation, and constitutive STAT3 activation independent of JAK2 mutations 61. JAK/STAT signaling plays a major role in many aspects of tumorigenesis, including proliferation, apoptosis, angiogenesis, and metastasis 62. Furthermore, JAK/STAT signaling helps establish and maintain the TIC phenotype in different cancer types, including breast cancer, non-small-cell lung cancer, endometrial cancer, colorectal cancer, and gliomas 61. For example, in HER2-overexpressing ER-positive human breast tumors, STAT3 phosphorylation via HER2/ER activation promoted a TIC phenotype 63. In this study, treatment with a STAT3 inhibitor suppressed the TIC phenotype, while knock-down of the STAT3 gene downregulated OCT4 expression and decreased tumorsphere formation 63. In breast cancer, OCT4 gene expression is regulated by IL-6 through IL-6-JAK1-STAT3 signaling, suggesting this pathway restores stemness properties by upregulating OCT4 expression 64. In pancreatic ductal adenocarcinoma, interleukin-22 (IL-22)-mediated activation of STAT3 promotes tumorsphere formation and invasion, and it increases the expression of pluripotency-associated transcription factors including SOX2 and NANOG as well as EMT markers. Conversely, treatment with an inhibitor of JAK/STAT signaling blocks these effects 65. Furthermore, knock-down of JAK2 in colorectal cancer cells leads to the decreased expression of stemness genes, including OCT4, SOX2 and NANOG, suggesting that JAK2/STAT3 signaling regulates stemness features via the expression of pluripotency-associated transcription factors 66.

Furthermore, the TIC phenotype in colorectal cancer can be regulated by an extracellular insulin-like growth factor (IGF) signaling pathway, which regulates NANOG via STAT3 phosphorylation 67. STAT3 signaling can also be activated by microRNA (miRNA), including miR-196b-5p, which targets negative regulators of STAT3 and leads to the upregulation of stem cell factors, including NANOG, OCT4, and SOX2 62. Lastly, colon cancer cells can be reprogrammed to a stem-cell-like state via STAT3 signaling. This study found that the CD44 c-terminus contributes to sphere formation by regulating integrin activation, and nuclear CD44/STAT3 signaling increases growth of tumor spheres as well as their expression of SOX2 and OCT4 68. These cells were more likely to colonize the lung when intravenously injected into mice, suggesting they are important drivers of metastasis.

### Wnt pathway

The Wnt pathway is a highly complex, evolutionarily conserved signaling pathway that includes many ligands and receptors 69. Signaling through this pathway can be distinguished as canonical, which is mediated by the transcriptional regulator β-catenin, or non-canonical, which is β-catenin-independent. Wnt signaling is engaged during development and has also been associated with carcinogenesis in various malignancies, including gastrointestinal cancers, leukemia, melanoma, and breast cancer. Wnt signaling contributes to the development of metastasis and the regulation of stemness features and tumor initiation 70. In MICs, the Wnt pathway is reported to upregulate proteins that inhibit differentiation, counteract apoptosis, and promote cell migration and invasion 61. In a non-small cell lung cancer cell line, Wnt/β-catenin signaling can control the transcript and protein levels of OCT4, and knock-down of β-catenin reduced OCT4 expression and mitigated a variety of stem-like properties *in vitro* 71. Additionally in non-small cell lung cancer, FOXC1 induces TIC properties by promoting β-catenin expression. In this study, β-catenin overexpression expanded the TIC sub-population, promoted tumorsphere formation, induced therapy resistance, and upregulated the levels of SOX2, OCT4, NANOG, and ATP-binding cassette transporter G2 (ABCG2), whereas β-catenin knock-down resulted in the opposite effects 72. In colorectal cancer, formation of a β‐catenin/TCF4:c‐JUN complex is a key nuclear mediator for NANOG activity, and this complex controls a sub-population of cells associated with the high expression of Wnt‐target genes 73. Indeed, resected mouse xenografts from this study showed that NANOG-GFP expressing cells had high levels of nuclear β-catenin, and these cells formed colony units *in vivo*. In gastric cancer cells, infection with *Helicobacter pylori* containing the CagA bacterial oncoprotein activated Wnt/β-catenin signaling via AKT and/or c-met mediated phosphorylation of β-catenin, which upregulated NANOG and OCT4 and increased stemness features 74. The Wnt pathway is associated with stemness and radiotherapy resistance in pancreatic carcinoma MICs, as HMGB1-TLR2/Wnt/β-catenin signaling regulated the expression of OCT4, SOX2, and NANOG 75. Interestingly, HMGB1 reduced stemness features upon binding an alternative receptor, TLR4, suggesting a balance of TLR2/TLR4 expression can regulate pancreatic cancer stemness. Additionally, in many cancer types OCT4 may activate Wnt/β-catenin signaling, suggesting a reciprocal regulation between these proteins to promote stemness 76.

### Notch pathway

The Notch pathway is an evolutionarily conserved signaling mechanism involved in the development of various organs during embryogenesis and in adult tissue homeostasis 77. The pathway is also involved in various malignancies and can act as an oncogene or as a tumor suppressor depending on factors such as signal strength, timing, cell type, and the tumor microenvironment 78.

Numerous studies have shown that activation of Notch promotes cell survival, self-renewal, and metastasis 61. In colon cancer, all Notch receptors, but particularly Notch2, regulate stemness in MICs. Notch2 had the highest expression in MICs compared with other Notch receptors, and suppression of this receptor decreased expression of OCT4 and SOX2 79. Additionally, Notch signaling is associated with SOX2 transcription, which seems to be reciprocal in some cancer types. For instance, hypoxia in ovarian cancer cells increases SOX2 promoter activity through Notch1 activation, which leads these cells to acquire stemness features including chemoresistance, tumorsphere formation, and the expression of specific cell-surface markers 80. Malignant glioma can feature a Notch1-SOX9-SOX2 positive-feedback loop that controls glioma stem cell invasion along white matter tracts. In this case, Jagged1-mediated Notch signaling activates SOX9, which then promotes SOX2 transcription 81. Furthermore, glioblastoma with TIC phenotype show upregulation of Notch1 signaling proteins NICD and Hes‑1, and knock-down of Notch1 in these cells diminishes their tumorsphere formation ability, self‑renewal, chemoresistance, and expression of OCT4, SOX2, and NANOG 82. Notch1 also appears to critically regulate stemness in head and neck squamous cell carcinoma (HNSCC) cells, as constitutive activation of NICD in HNSCC cells promotes self-renewal by enhancing tumorsphere formation and upregulating stemness markers such as OCT4, SOX2, and CD44. In contrast, Notch1 knock-down in primary HNSCC cells attenuated their TIC traits, sensitized them to chemotherapy and inhibited tumor formation in a mouse xenograft model*.* Notably, Notch1 acted upstream of canonical Wnt signaling in HNSCC cells 83. Finally, Notch1 is involved in the regulation of stemness in thyroid cancer cells 84. This study found that Notch1 knock-down led to downregulation of proteins related to stemness and EMT markers, including Notch1, SOX2, OCT4, and NANOG, and decreased cell viability and migration 84.

In summary, both the TIC phenotype and the expression of OCT4, SOX2, and NANOG are dictated by a collective of signaling pathways, all of which have been previously established as pro-tumorigenic. While this review highlights the progress in revealing the connection between these signaling pathways to the TIC phenotype, further studies are required to fully understand the complex mechanism in which these signaling pathways lead to the expression of pluripotency-associated transcription factors. Developing a full understanding of how components of these signaling pathways establish the TIC phenotype will provide a unique opportunity to discover promising means of targeting MICs.

## Chemokines and chemokine receptors that regulate pluripotency genes

Chemokines are small cytokines that direct immune cells to migrate to specific organs and regulate inflammation and immunity. These proteins are also involved in many other biological processes, including cell proliferation, survival, and differentiation. Chemokines exert their effects by binding to chemokine receptors, and are divided into four subfamilies (CC, CXC, CX3C, and XC) based on the configuration of the two cysteines closest to the N terminus. Chemokine receptors are also divided into four groups based on the subfamilies of chemokines they bind 85. Conventional chemokine receptors are seven-transmembrane G protein-coupled receptors (GPCRs) that typically transduce signals through Gα_i_ pathways, although some receptors couple to Gα_q_ family members 85,86. Chemokines are well-documented contributors to tumor progression as they recruit leukocytes such as regulatory T cells (Tregs), and promote angiogenesis, tumor growth and proliferation, and metastasis. As a result, many chemokine receptor inhibitors have been designed as therapeutics for cancer 87. Furthermore, numerous studies suggest that chemokine/chemokine receptor pairs regulate the TIC phenotype. This is consistent with the fact that chemokines signal through a complex network of pathways, many of which appear to play crucial roles in tumor initiation. In turn, several chemokine receptors are upregulated during tumorsphere formation, and several of these receptors are confirmed to regulate stemness features 88. Some chemokine receptors have been described as MIC markers, including CXCR1/2 and CXCR4 89 (please see also Table 1).

## CXCR subfamily

### CXCR1/2

The chemokine receptors CXCR1 and CXCR2 share considerable structural similarity, as 78% of their amino acid sequences are identical 90. Both receptors can couple to the same Gα_i_ and Gα_q_ GTP binding proteins and are present in the same cells, but they have unique ligand specificity. While CXCR1 only binds to two ligands (CXCL6 and CXCL8), CXCR2 is less selective, and its known ligands include CXCL1, CXCL2, CXCL3, CXCL5, CXCL6, CXCL7, and CXCL8 86. Both CXCR1 and CXCR2 are expressed on granulocytes, monocytes, mast cells, and NK cells, and can also be expressed on cancer cells 91,92.

The chemokine ligands for CXCR1/2 are expressed and secreted by different cancer cell types, and these ligands stimulate proliferation and migration by acting in an autocrine fashion 92. The most potent ligand for both CXCR1 and CXCR2 is CXCL8, also known as interleukin-8 (IL-8) 90,93. IL-8 is a pro-inflammatory CXC chemokine produced by various cell types that recruits neutrophils to sites of infection or tissue injury 92. IL-8-receptor binding induces a multitude of pathways. Of great importance are the activation of PI3K and phospholipase C, which promote the activation of AKT, PKC, calcium mobilization, and/or MAPK signaling cascades. These pathways enhance cell survival and migration in both neutrophils and cancer cells 91. IL-8 signaling also promotes the nuclear translocation of STAT3 and β-catenin and activates RhoGTPases and tyrosine kinases such as Src and focal adhesion kinase (FAK) that regulate the cytoskeleton and mediate cell motility and invasion 94.

IL-8 and its receptors are expressed by cancer cells as well as infiltrating immune cells, and IL-8 signaling in both cell types is involved in angiogenesis, proliferation, survival, migration, and chemotherapeutic resistance 94. Furthermore, the IL-8-CXCR1/2 axis regulates stemness features in MICs of various cancer types, including lung cancer 95, pancreatic cancer 96, breast cancer 97, and hepatocellular carcinoma 98. Breast cancer MICs express CXCR1 and CXCR2, and both receptors help to establish and maintain a stemness phenotype and induce EMT, thus contributing to metastasis 99. As expected, the IL-8-CXCR1/2 axis also regulates the expression of pluripotency-associated genes. In small-cell lung cancer, IL-8 transcript expression and protein secretion is increased in MICs, and this chemokine acts in an autocrine fashion to induce stemness features including self-renewal, migration, tumorsphere formation, and the expression of stemness related genes 100. Knock-down of IL-8 in these MICs led to a decrease in OCT4 and NANOG expression as well as an inhibition of tumor growth in a mouse xenograft model, while treatment with exogenous recombinant human IL-8 upregulated SOX2 and NANOG *in vitro* 100. CXCR1/2 and their ligands are both expressed by lung cancer cells, and receptor inhibition with an antagonist decreased migration and enhanced apoptosis *in vitro* and suppressed tumor growth, metastasis, and angiogenesis in a mouse xenograft model 101. Resected lung cancer xenografts from this study provided information on how the antagonist repressed important molecular pathways, including ERK1/2 and AKT pathways as well as the expression of NF-κB p65 and vascular endothelial growth factor (VEGF).

In colorectal cancer, both CXCR1 and IL-8 are overexpressed in patients' diseased colon tissue specimens, and their expression, in conjunction with the stemness marker aldehyde dehydrogenase 1 (ALDH1), was correlated with poor survival 102. This study also noted that knocking down CXCL8 or CXCR1 from primary colon cancer isolates decreased their proliferation and angiogenesis *in vitro* and in a secondary xenograft model, which was associated with the dysregulation of several cell cycle proteins. Also, in primary colorectal cancer cells/tissues the EMT activator Snail was overexpressed in stem-like cells and promoted the expression of several hundred Snail-activated genes, which notably include IL-8 103. Snail bound directly to the IL-8 promoter E3/E4 E-boxes to facilitate IL-8 expression, and deletion or mutagenesis of select residues in these regions mitigated IL-8 expression *in vitro* 103. Snail overexpression correlated with self-renewal and chemoresistance, and these properties were reverted when IL-8 activity was blocked using a neutralizing antibody. Furthermore, both shRNA-mediated IL-8 knock-down and IL-8 neutralization decreased the expression of SOX2, NANOG, and OCT4 in MICs 103. Notably, Snail can be expressed in primary tumors and metastases, and its expression is regulated by an integrated and complex signaling network that includes PI3K/AKT, TGF-β, Notch, Wnt and NF-κB pathways 104.

CXCR2 is also overexpressed in GICs, and IL-8 increases the self-renewal capacity of glioblastoma cells and the expression of GIC markers *in vitro* and enhances tumor growth and therapy resistance *in vivo* 105. Specifically, IL-8 activation of CXCR2 in glioblastoma cell lines significantly increased SOX2 and NANOG expression and immunoblot analysis of tumor cell lines obtained from patient-derived xenografts (PDXs) and exposed to IL-8 showed a time-dependent increase in the expression of critical GIC-associated transcription factors, including NANOG, SOX2, and OCT4 105.

In addition to IL-8, other chemokine ligands for CXCR1/2 can regulate stemness, although this depends on the specific ligand and cancer type. For instance, a study in thyroid cancer compared how two CXCR2 ligands, CXCL1 and IL-8, regulate stemness features. Only IL-8 was crucial for self-renewal, tumorsphere formation and tumor initiation capabilities as well as the expression of the stemness markers OCT4, SOX2, and NANOG. IL-8 expression was also correlated with lymph node metastasis 106. In contrast, colon cancer cells treated with CXCL1 showed increased tumor-initiating properties, including tumorsphere formation and the expression of OCT4, NANOG, SOX2 and other stemness markers *in vitro* 107. Another CXCR2 ligand, CXCL3, may regulate stemness features in hepatocellular carcinoma, including proliferation, self-renewal, and tumorigenesis *in vivo* 108. CXCL3 was significantly overexpressed in a CD133^+^ sub-population within hepatocellular carcinoma, and siRNA silencing of CD133 reduced tumor weight in a mouse xenograft model and led to reduced expression of CXCL3 *in vitro*, demonstrating a positive correlation between the two proteins 108. Exogenous CXCL3 treatment increased the expression of OCT4 and other stemness-related genes including EP300, Tert, and β-catenin, while CXCL3 knock-down downregulated these same genes. The authors of the study proposed that CXCL3 may regulate stemness features through the MAPK pathway by activating ERK1/2, which phosphorylates ETS1 108. Furthermore, high CXCR2 expression has also been reported in hepatocellular carcinoma. One study demonstrated that CXCR2 expression correlates with intrahepatic metastasis and decreased differentiation but no other factors like age and gender, AFP levels, tumor capsule, or tumor size 109. Finally, CXCR2 signaling in renal cell carcinoma can be regulated by galectin-3 (Gal-3), which in turn regulates the stemness features of renal MICs 110. This study showed that Gal‐3 silencing via shRNA inhibited CXCL6- and CXCL7-mediated CXCR2 signaling in MICs and simultaneously inhibited the expression of NANOG and SOX2, therefore suppressing the stemness phenotype of these cells. Interestingly, CXCR2 overexpression restored NANOG and SOX2 expression as well as the stem-like features in Gal‐3-silenced MICs. Furthermore, overexpression of Gal‐3 in renal carcinoma cells promoted *in vivo* tumorigenicity in a mouse xenograft model 110.

### CXCR3

CXCR3 is a chemokine receptor expressed on monocytes, T cells, NK cells, and dendritic cells (DCs), and its ligands include CXCL11, CXCL10, CXCL9, and CXCL4. The receptor plays roles in inflammation, wound healing, and immunity, and additionally in both autoimmune diseases and tumor progression 111.

CXCR3 has two major isoforms formed by alternative splicing, CXCR3A and CXCR3B. CXCR3A expression was previously implicated in metastasis, as it promotes cell migration and invasion in various cancers, including colorectal and gastric 112,113. In contrast, CXCR3B appears to regulate stemness. In breast cancer, CXCR3B is upregulated in MICs, and its overexpression increases the number of these cells, while its silencing has the opposite effect 114. In addition, small molecule inhibition of CXCR3 blocks breast cancer cells from colonizing the lung when injected intravenously in mice. This regulation seems to occur through CXCR3 ligand-mediated activation of STAT3, ERK1/2, CREB, and NOTCH1 pathways 114. Furthermore, the CXCR3 ligand CXCL11 was upregulated in hepatocellular carcinoma MICs and induced the expression of stem cell-related genes, including NANOG. In this study, CXCL11 signaling through CXCR3 led hepatocellular carcinoma cells to acquire and maintain properties like self-renewal, tumorigenicity, and chemoresistance via activation of the ERK1/2 pathway, and CXCL11 knock-down reduced tumorgenicity in a mouse xenograft model 115.

### CXCR4

**Figure 2:**
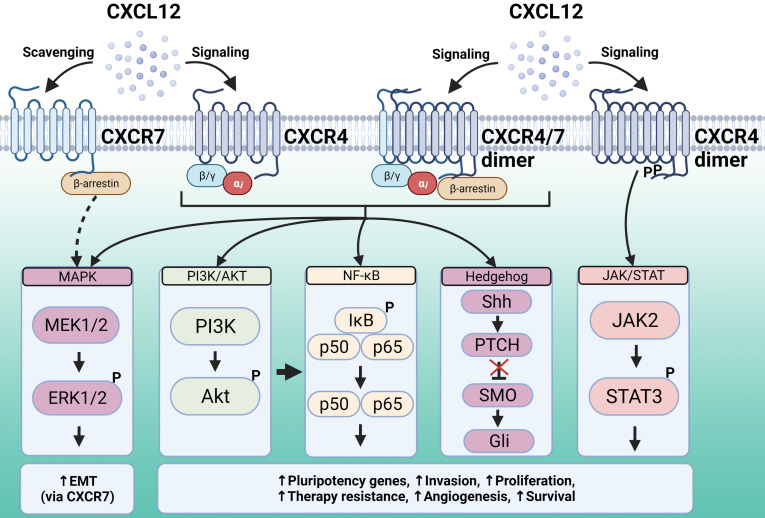
The chemokine CXCL12 is engaged by two different receptors, CXCR4 and CXCR7. CXCR4 downstream signaling regulates gene transcription, chemotaxis, cell survival, and proliferation. The signaling pathways activated upon CXCL12 binding to CXCR4 and subsequent recruitment of G proteins include PI3K-AKT, NF-κB and MAPK, whereas the JAK/STAT signaling pathway is recruited in a G-protein-independent manner. All these pathways support malignancy and stemness features. Unlike typical GPCRs, CXCR7 is generally not coupled to G proteins. This receptor can activate MAPK effectors via β-arrestin, whereas in some cell types it can form heterodimers with CXCR4 and act as a co-receptor. Additionally, CXCR7 has been proposed to scavenge excess CXCL12 through high-affinity binding and degradation, thus modulating ligand binding to CXCR4. Created with Biorender.com.

CXCR4 is expressed by most hematopoietic cell types, and its main ligand is CXCL12, also known as stromal cell-derived factor 1 (SDF-1). CXCR4 plays a role not only in leukocyte recruitment, but also in crucial processes during embryogenesis, including the development of the hematopoietic, cardiovascular, and nervous systems 116. This chemokine receptor is also involved in pathologies including human immunodeficiency virus (HIV) infection, where it acts as a HIV co-receptor, as well as cancer and immune diseases. CXCR4 activation and subsequent signal transduction regulates gene transcription, chemotaxis, cell survival, and proliferation. CXCR4 signaling pathways activated by ligand binding include PI3K-AKT-NF-κB, MEK1/2, and ERK1/2, as well as JAK/STAT signaling in a G-protein-independent manner 117 (**Figure 2**).

CXCR4 is overexpressed in multiple cancer types and contributes to tumor growth, invasion, migration, metastasis, relapse, and therapy resistance 117. Furthermore, CXCR4 has been previously associated with the regulation of the TIC phenotype. In renal cell carcinoma cells, CXCR4 is preferentially expressed in cells with high expression of OCT4, SOX2, and NANOG. These CXCR4 expressing cells had increased therapy resistance, tumorsphere formation and tumor growth capabilities compared to cells that lacked CXCR4 expression, and CXCR4 knock-down by siRNA mitigated these stemness features 118. CXCR4 is also involved in chemotherapy resistance in colorectal cancer cells. Oxaliplatin-resistant colorectal cancer cells strongly expressed CXCR4, and *in vitro* treatment with a CXCR4 antagonist mitigated treatment resistance and the downstream pathways involved, including PI3K/AKT and ERK1/2 intracellular signaling cascades and the NF-κB transcription factor 119. Similarly, upregulation of the CXCL12/CXCR4 axis was reported in esophageal MICs, and this signaling axis maintained a TIC phenotype in these cells via activation of the ERK1/2 pathway 120. This study demonstrated that CXCR4 and ERK1/2 activation enhanced esophageal cancer cell line migration and invasion in transwell and Matrigel models, which was blocked by antagonists of both proteins or CXCR4 silencing. A study in non-small cell lung cancer showed that CXCR4 expression was higher in a drug-resistant cell line compared with the parental cell line, suggesting that this chemokine receptor is involved in drug resistance 121. The authors used fluorescence activated cell sorting (FACS) to isolate cells expressing and cells lacking CXCR4, which revealed that CXCR4 expressing cells had significantly higher levels of OCT4, SOX2, and NANOG. CXCR4 expressing cells also displayed higher self-renewal potential, drug resistance, and tumorigenic potential in a mouse xenograft model. This study also showed that CXCR4 expression was regulated by the PI3K/AKT/mTOR pathway, while the CXCR4 downstream effector STAT3 seemed to regulate stemness features 121.

MICs may use CXCR4 to maintain their stemness features via regulation of pluripotency-associated genes. In GICs, various studies have described upregulation of the CXCL12/CXCR4 pathway as well as CXCR4-mediated regulation of pluripotency-associated genes, proliferation, invasion, angiogenesis, and therapy resistance 122-125. For instance, CXCR4 is essential for the self-renewal of glioblastoma GICs, since disruption of CXCL12/CXCR4 signaling and its downstream ERK and AKT pathways reduced expression of OCT4 and NANOG *in vitro* 123. This was further supported by a study in human patient specimens that showed CXCR4 is co-expressed with OCT4, SOX2, and NANOG in both primary and recurrent glioblastomas 124. Additionally, glioblastoma cells treated with a small-molecule CXCR4 inhibitor showed decreased expression of TIC markers, including SOX2 and NANOG 122. This same inhibitor also increased apoptosis, reduced CXCR4 expression and cell migration *in vitro*, and impaired tumor initiation in a mouse subcutaneous xenograft model as well as a mouse model where glioblastoma cells were directly injected into the brain 122. Furthermore, in glioblastoma the inhibition of CXCR4 and CXCL12 by a miRNA cluster blocked tumor development in mice with cancer cells implanted into the striatum and decreased the expression of OCT4 and NANOG *in vitro*. The authors suggested that NANOG expression was regulated by Hh signaling, and that a functional CXCR4 pathway was necessary to maintain a Hh-Gli1-NANOG network and self-renewal properties 125. CXCR4/CXCL12 signaling also activates the Hh pathway in other cancer types, thus increasing tumor size, cell motility, angiogenesis, EMT, cell invasion, and NANOG expression 126. Interestingly, there appears to be a mutual regulation of CXCR4 and NANOG in stem-like cancer cells, which could be mediated by PI3K/Akt/NF-κB and SHH/Gli1 pathways 126.

### CXCR7

CXCR7 is a seven transmembrane-spanning chemokine receptor that, unlike typical GPCRs, is generally not coupled to Gαi proteins. It is an atypical chemokine receptor (ACKR3) that functions through G-protein-independent mechanisms in most cell types 127. CXCR7 has multiple ligands, although the most widely studied is the chemokine CXCL12, which is also a ligand for CXCR4 127. The mechanisms of signal transduction through this receptor are not fully known, but evidence suggests that in some cell types, CXCR7 forms heterodimers with CXCR4 to act as a co-receptor (**Figure 2**). CXCR7 can also signal via β-arrestin, which activates MAPK effectors 128. Additionally, CXCR7 has been proposed to scavenge excess CXCL12 through high-affinity binding and degradation, which modulates ligand binding to CXCR4 128.

Like CXCR4, CXCR7 can mediate various cancer processes, including tumor growth and metastasis. However, CXCR7 and CXCR4 appear to be co-expressed in some cancers (i.e breast cancer lines like MDA-MB-231 and MCF-7 129), but not in others (recurrent glioblastoma 124), suggesting that only MICs from select types of cancer utilize CXCR7 functions. In primary and recurrent glioblastoma patient samples, CXCR4 but not CXCR7 expression was correlated with the expression of pluripotency-associated genes 124. In contrast, studies in other cancer types have reported that CXCR7 can regulate stemness and the expression of pluripotency transcription factors. In lung cancer, CXCR7 expression is upregulated by TGF-β1 signaling, and this promotes motility, invasion, EMT, and an increase in TIC features in cancer cells 130. TGF-β1 signaling also upregulated other chemokine receptors, including CXCR4, although to a lesser extent. Upregulation of these chemokine receptors led to an increase in the transcript and protein levels of OCT4, SOX2, and NANOG, which was reversed by silencing CXCR4 and/or CXCR7. CXCL12 was also upregulated by TGF-β1 activation, and its binding to CXCR4 and/or CXCR7 was a crucial factor to promote expression of stemness genes. However, only CXCR7 was essential for EMT, and high expression of TGF-β1 and CXCR7 was correlated with poor patient survival. The authors proposed that TGF-β1-induced upregulation of CXCR7 occurs via the Smad pathway 130. In breast cancer, CXCR7 is significantly overexpressed in CD44^+^/CD24^low^ MICs. Furthermore, knocking down CXCR7 resulted in a significant loss of the CD44^+^/CD24^low^ phenotype, along with decreased tumorsphere formation and reduced protein levels of OCT4 and NANOG. Silencing CXCR7 also inhibited tumor growth in a mouse xenograft model and sensitized cells to chemotherapy by increasing apoptosis 131. Similarly, overexpressing the chemokine CXCL12 in a breast cancer cell line led to an upregulation of OCT4, SOX2, and NANOG, as well as an increase in stemness features and EMT markers 129. CXCL12 treatment led to the nuclear translocation of p65 and increased IkB phosphorylation, suggesting an activation of the NF-kB pathway, and blocking this pathway impaired CXCL12-induced EMT 129.

### CX3CR1

**Figure 3:**
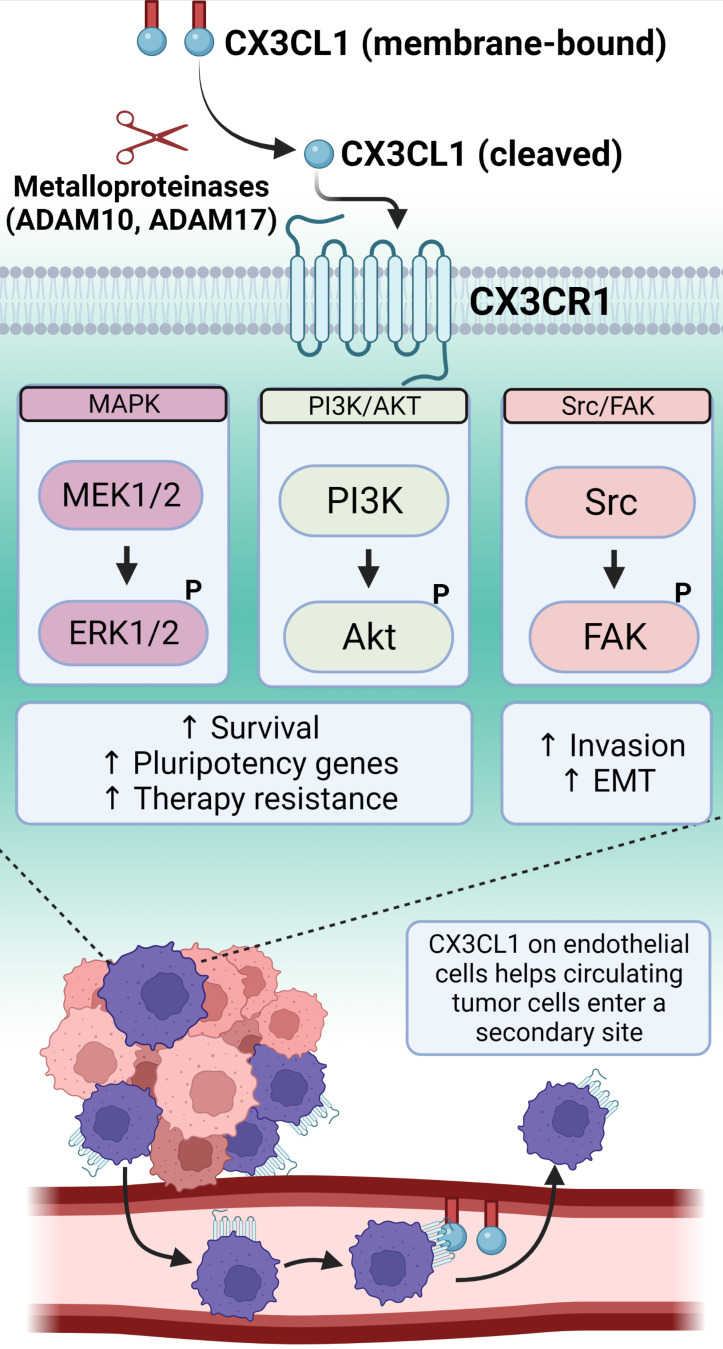
CX3CR1 binds to a single chemokine ligand, CX3CL1, which exists in a membrane-bound form that can be cleaved by metalloproteinases into a soluble molecule. CX3CR1 downstream signaling involves several pathways important for promoting a TIC phenotype. The binding of CX3CR1 with the membrane-bound form of CX3CL1 results in cell-adhesive interactions. This allows cancer cells traveling through the systemic blood circulation to arrest at distant sites and then extravasate following the chemoattractant gradient generated by the soluble CX3CL1. Created with Biorender.com.

The chemokine receptor CX3CR1 is a seven transmembrane G-protein coupled receptor with a single known ligand, fractalkine (CX3CL1), and it is notably important for leukocyte adhesion and migration 132. CX3CL1 can be expressed as a transmembrane protein that mediates cellular adhesion, and it can also be cleaved by metalloproteinases to produce a soluble protein that mediates chemoattraction 133-135 (**Figure 3**). Recently, numerous studies have implicated CX3CR1 in cancer progression. In ovarian cancer, CX3CR1 is generally overexpressed, and the degree of overexpression correlates with cancer stage 136. Follow up studies in ovarian cancer cell lines showed that hypoxia upregulated CX3CR1, and these cells responded to CX3CL1 in cell spheroid invasion assays. In select lung cancer cell lines, CX3CR1 signals through the Src/FAK axis to promote both migration and invasion in transwell and Matrigel assays, and this was blocked by saracatinib, a small molecule inhibitor of Src family kinases 137. In breast cancer, CX3CR1 expression correlates with brain metastasis 138, and CX3CR1 is also expressed within spinal metastases and acts through the Src/FAK signaling axis to promote migration and invasion 139. When breast cancer cells are injected into the left ventricle of mice, CX3CR1 drives cancer cells to the skeleton through interactions with CX3CL1 expressed by bone marrow endothelial cells 140. This skeletal seeding can be inhibited by a small-molecule antagonist of CX3CR1, which also impairs metastatic progression in the same model 141. CX3CR1 is expressed on circulating tumor cells, and inhibiting this receptor keeps the cells in systemic circulation, which promotes apoptosis 142. Prostate cancer spinal metastases express high levels of CX3CR1, and overexpression of CX3CR1 in prostate cancer cell lines promotes proliferation, migration, and invasion while inhibiting apoptosis 143. Follow up studies in prostate cancer cell lines show that they express CX3CR1, the receptor enhances migration in a scratch wound assay, and migration is dampened by CX3CR1 knock-down or by inhibitors of the Src/FAK pathway. Like breast cancer cells, CX3CR1 expression by prostate cancer cells drives their adherence to bone marrow endothelial cells 144, and malignant transformation is associated with increased expression of CX3CR1 145 (**Figure 3**).

Recently, we reported that human tissue specimens of prostate and breast tumors harbor cells that express high levels of CX3CR1 (CX3CR1^High^), and these CX3CR1^High^ cells co-express OCT4 and NANOG 146. Additionally, human prostate and breast cancer cell lines contain a sub-population of CX3CR1^High^ cells, with CX3CR1 expression consistently correlating with high OCT4 and NANOG expression in comparison to cells with low CX3CR1 expression (CX3CR1^Low^) 146. We further demonstrated that CX3CR1^High^ cells were enriched for stemness features using global transcriptome analysis 146. When pure populations of CX3CR1^High^ or CX3CR1^Low^ cells were injected via the intracardiac route in an animal model of metastasis, the CX3CR1^High^ cells showed the highest propensity to initiate and develop metastatic lesions 146. Furthermore, whole transcriptome analysis revealed that CX3CR1^High^ cells were de-enriched for the Hallmark apoptosis signature 146. High CX3CR1 status resulted in resistance to docetaxel treatment, which is the standard-of-care chemotherapy for breast and prostate cancer 146. The crucial findings of this study indicate that CX3CR1^High^ cells have a stemness phenotype that includes resistance to chemotherapy. Additionally, CX3CR1^High^ cells are endowed with metastasis-initiating ability in both prostate and breast cancer. We showed that lung cancer and melanoma cell lines also harbor CX3CR1^High^ sub-populations and have high levels of OCT4 and NANOG, indicating that CX3CR1 may be a pan-cancer marker of MICs 146.

## CCR subfamily

### CCR4

CCR4 is the receptor for two chemokine ligands: CCL17 and CCL22. This chemokine receptor is predominantly expressed by Th2 cells, cutaneous lymphocyte antigen-positive skin-homing T cells, and Treg cells. CCR4-expressing T cells are guided into inflamed skin by CCL17 and CCL22, and thus, the receptor regulates skin T cell homing and cutaneous inflammation 147.

CCR4 expression is also associated with tumor progression. High CCR4 expression in the tumor microenvironment corresponds with high levels of Tregs, which can generate an immunosuppressive microenvironment and facilitate the immune escape of malignant cells. In turn, overexpression of CCR4 has been associated with poor prognosis and decreased survival in different cancers 148,149. Aberrant CCR4 expression in tumor cells has been associated with tumor progression and metastasis in breast cancer 150, hepatocellular carcinoma 151, and oral tongue cancer 152. Recently, the expression of this receptor and its ligands were found to induce a TIC phenotype. Hepatocellular carcinoma cells express high levels of CCR4 153, and treating these cells with either the conditioned medium from M2 macrophages, which express higher CCL17 levels than M1 macrophages, or with exogenous CCL17, promoted survival, migration, and tumorsphere formation. Furthermore, both treatments increased the relative levels of NANOG, OCT4, and SOX2 and enhanced Wnt/β-catenin activation 153. The authors suggested that the Wnt/β-catenin pathway downstream of CCL17/CCR4 signaling regulated the stemness of hepatocellular carcinoma cells. Notably, co-implantation of hepatocellular carcinoma cells with M2 macrophages significantly increased tumor growth in a mouse xenograft model compared to co-implantation with other types of cells, indicating that CCL17 can drive tumorigenicity 153.

### CCR7

CCR7 is expressed by naïve lymphocytes, central memory T cells, and mature DCs. Its ligands, CCL19 and CCL21, are expressed by stromal cells within lymphoid organs, and are considered homeostatic chemokines as they direct cell recruitment under resting conditions 154. This receptor/ligand axis plays an essential role in the migration of T cells towards lymphoid organs and DC homing to lymph nodes for antigen presentation 154.

CCR7 expression has been detected in breast cancer, non-small cell lung cancer, head and neck cancer, colorectal cancer, stomach cancer, melanoma, chronic lymphocytic leukemia, non-Hodgkin's lymphoma, and T cell leukemia 154. Expression of this receptor has been associated with tumor aggressiveness, metastasis, and decreased patient survival 155. Furthermore, multiple studies have implicated the overexpression of this receptor/ligand pair in the TIC phenotype. In pancreatic cancer, CCR7 expression was significantly increased in MICs as well as resected primary tumors and metastatic lymph nodes, and CCL21/CCR7 signaling promoted metastasis, EMT, and survival by modulating the ERK1/2/NF-κB pathway 156. In colorectal cancer, CCR7 expression was associated with cancer progression, metastasis to the lymph nodes, and decreased patient survival 157,158. *In vitro* studies of colorectal cancer cells showed that treatment with CCL21 upregulated P-glycoprotein and NANOG/OCT4, which enhanced chemotherapy resistance and promoted cell survival and tumorsphere formation, respectively 159. The authors showed that the CCR7-mediated regulation of TIC features and pluripotency transcription factors required the EMT protein Snail, and that Snail was upregulated by CCL21 via PI3K/AKT/GSK-3β signaling 159. Additionally, this receptor/ligand axis has been implicated in the regulation of stemness features in oral squamous cell carcinoma (OSCC) 160. CCR7 and its ligand CCL21, but not CCL19, were overexpressed in diseased oral tissues from OSCC patients, and CCR7 expression correlated with a poor prognosis 160. Studies in OSCC cell lines showed that exogenous CCL21 stimulation upregulated OCT4 and other TIC markers and enhanced EMT and stemness features, including migration, invasion, tumorsphere formation, and colony formation. CCL21 treatment increased the phosphorylation of JAK2 and STAT3, while treatment with a JAK2 inhibitor suppressed both CCL21-induced EMT and the TIC phenotype 160. These results suggest that the CCL21/CCR7 axis regulates EMT and the stemness of OSCC cells through the activation of the JAK2/STAT3 pathway.

It is crucial to establish the master regulator transcription factors and signaling pathways that confer a TIC phenotype as a potential means of therapeutically targeting this small group of cells. The evidence that chemokine receptors can both identify and drive the TIC phenotype is a fundamental finding, as the surface expression of chemokine receptors can be used to isolate sub-populations of MICs. Chemokine receptors have been widely studied in immune cells, and there are good reagents and protocols available to study these receptors. By using FACS to isolate MICs based on the surface expression of chemokine receptors, pure sub-populations of MICs can be further characterized at the molecular level.

## Conclusions

Together, the master regulator transcription factors OCT4, NANOG, and SOX2 form a core that establishes the pluripotent state in embryonic stem cells. This is achieved by increasing the expression of pluripotency factors, including themselves, and by repressing genes that encode lineage-specific factors 161. Co-expression of these master regulator transcription factors has been found in a wide range of cancer types, especially in poorly differentiated tumors, where they control the fate of MICs during cancer progression 162. In addition, several studies have demonstrated that increasing the expression of these pluripotency transcription factors in non-MICs leads them to acquire a TIC phenotype 162. Given that OCT4, SOX2 and NANOG are crucial to maintain stemness, we need a greater knowledge of the mechanisms that regulate the expression of these factors to design new therapeutic interventions that can eventually overcome issues such as chemotherapy resistance, tumor recurrence, and metastasis. This review summarizes the various signaling pathways that activate these master regulator transcription factors, thus generating the TIC phenotype. With chemokine receptors upstream of these signaling pathways and master regulator transcription factors, further studies are needed to characterize their role in tumor initiation and cancer cell stemness. This newly acquired knowledge will be paramount to pursue chemokine receptors as therapeutic targets.

## Figures and Tables

**Table 1:**
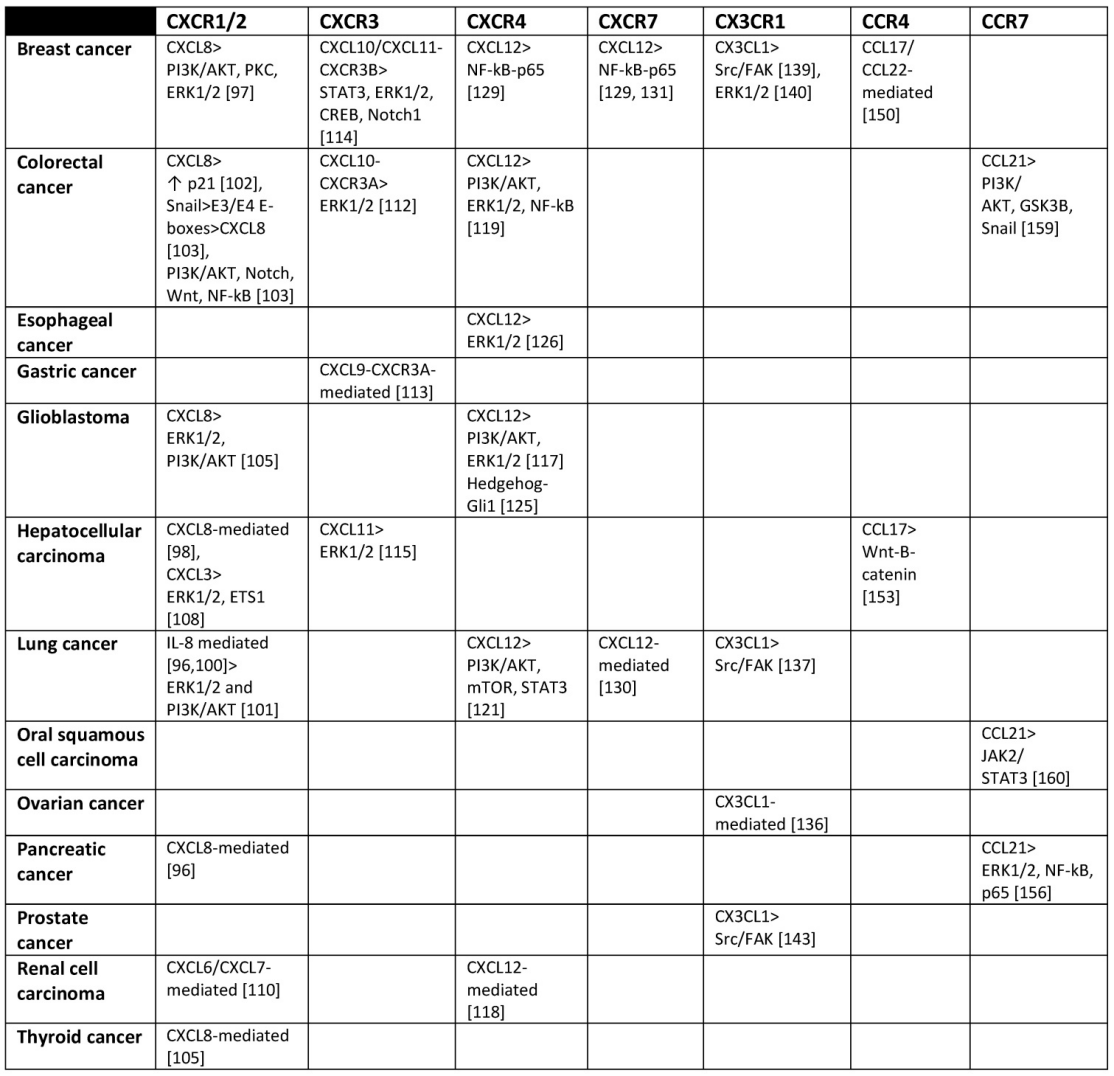
Chemokine receptors and cellular signaling pathways they activate that have been found associated with MICs.

## Abbreviations

PI3K: phosphoinositide 3-kinase
AKT: alpha serine/threonine protein kinase
TGF-β: transforming growth factor beta
EGFR: epidermal growth factor receptor
JAK: janus kinase
STAT: signal transducer and activator of transcription
HER2: human epidermal growth factor 2
ER: estrogen receptor
EMT: epithelial mesenchymal transition
FOXC1: forkhead box C1
MAPK: mitogen-activated protein kinase
ERK: extracellular signal regulated kinase
CREB: cAMP response element binding protein
MEK: mitogen-activated kinase
FAK: focal adhesion kinase
